# Deregulation of microRNA expression in monocytes and CD4^+^ T lymphocytes from patients with axial spondyloarthritis

**DOI:** 10.1186/s13075-019-1829-7

**Published:** 2019-02-12

**Authors:** Olivier Fogel, Andreas Bugge Tinggaard, Maud Fagny, Nelly Sigrist, Elodie Roche, Laurence Leclere, Jean-François Deleuze, Frederic Batteux, Maxime Dougados, Corinne Miceli-Richard, Jörg Tost

**Affiliations:** 1 Laboratory for Epigenetics and Environment, Centre National de Recherche en Génomique Humaine, CEA - Institut de Biologie François Jacob, 2 rue Gaston Crémieux, Evry, France; 2 Department of Rheumatology - Hôpital Cochin. Assistance Publique - Hôpitaux de Paris, Paris Descartes University, Paris, France; 3 Department of Biomedicine, Aarhus University, Aarhus, Denmark; 4 Department of Immunology, Cochin Hospital, Paris, France; 5 Unité Mixte AP-HP/ Institut Pasteur, Institut Pasteur, Immunoregulation Unit, Paris, France; 6 INSERM (U1153) : Clinical Epidemiology and Biostatistics, PRES Sorbonne Paris-Cité, Paris, France

**Keywords:** Spondyloarthritis, MiRNA, Epigenetics, CD4^+^ T lymphocytes, Monocytes

## Abstract

**Background:**

MicroRNAs (MiRs) play an important role in the pathogenesis of chronic inflammatory diseases. This study is the first to investigate miR expression profiles in purified CD4^+^ T lymphocytes and CD14^+^ monocytes from patients with axial spondyloarthritis (axSpA) using a high-throughput qPCR approach.

**Methods:**

A total of 81 axSpA patients fulfilling the 2009 ASAS classification criteria, and 55 controls were recruited from October 2014 to July 2017. CD14^+^ monocytes and CD4^+^ T lymphocytes were isolated from peripheral blood mononuclear cells. MiR expression was investigated by qPCR using the Exiqon Human MiRnome panel I analyzing 372 miRNAs. Differentially expressed miRNAs identified in the discovery cohort were validated in the replication cohort.

**Results:**

We found a major difference in miR expression patterns between T lymphocytes and monocytes regardless of the patient or control status. Comparing disease-specific differentially expressed miRs, 13 miRs were found consistently deregulated in CD14^+^ cells in both cohorts with miR-361-3p, miR-223-3p, miR-484, and miR-16-5p being the most differentially expressed. In CD4^+^ T cells, 11 miRs were differentially expressed between patients and controls with miR-16-1-3p, miR-28-5p, miR-199a-5p, and miR-126-3p were the most strongly upregulated miRs among patients. These miRs are involved in disease relevant pathways such as inflammation, intestinal permeability or bone formation. Mir-146a-5p levels correlated inversely with the degree of inflammation in axSpA patients.

**Conclusions:**

We demonstrate a consistent deregulation of miRs in both monocytes and CD4^+^ T cells from axSpA patients, which could contribute to the pathophysiology of the disease with potential interest from a therapeutic perspective.

**Electronic supplementary material:**

The online version of this article (10.1186/s13075-019-1829-7) contains supplementary material, which is available to authorized users.

## Background

Axial spondyloarthritis (axSpA) is a chronic inflammatory rheumatic disease that mainly affects the spine and sacroiliac joints in young adults. AxSpA includes both radiographic axSpA, also known as ankylosing spondylitis (AS), characterized by radiographic sacroiliitis according to the modified New York criteria [1], and non-radiographic axSpA (nr-axSpA), characterized by the absence of structural damage of the sacroiliac joints on X-rays. The disease is frequently associated with extra-articular manifestations including psoriasis, uveitis, or inflammatory bowel disease [2, 3]. The long-term outcome of AxSpA is determined by structural damages such as the formation of syndesmophytes, excessive new bone formation of the spine [4].

Strong evidence suggests that disease pathogenesis is driven by innate immune cells (monocytes/macrophages, dendritic cells) and CD4^+^ T lymphocytes [3, 5–8]. Genetic variation associated with AxSpA was found enriched in open chromatin regions in monocytes and CD4^+^ T lymphocytes, suggesting these cell populations might play an important role in the disease [9]. Genetic analyses identified the *HLA-B27* and *IL23R* loci, whose proteins are present at the cell membrane of monocytes and T lymphocytes respectively, as the genetic factors most strongly associated with the disease [10, 11]. Monocytes/macrophages are crucially involved in the disease pathogenesis notably through the HLA-B27-induced unfolded protein response (UPR) stress leading to the release of pro-inflammatory cytokines (IL-1α, TNF, IL-6, and IL-23) [12]. Furthermore, T cells and macrophages have been found in the cellular infiltrates of tissue biopsies from AS patients [13–18]. Adoptive transfer studies from disease-prone B27 transgenic rats to B27 nude rats demonstrated that CD4^+^ T cells were able to induce the disease [7].

MicroRNAs (miRs) are small non-coding RNAs composed of 18–25 nucleotides that play an important regulatory role at the post-transcriptional level in diverse biological processes including cell differentiation, proliferation, apoptosis, or cellular function [19]. MiRs are involved in immune functions during granulopoiesis, T and B cell ontogenesis, TLR signaling, and cytokine production [20, 21]. We have previously shown that miR expression profiles are highly cell type specific with different expression profiles observed between CD4^+^ T and CD19^+^ B lymphocytes [22], reflecting their specific roles in a broad range of cellular functions. While miR deregulation has been comprehensively investigated in several autoimmune or rheumatic diseases including rheumatoid arthritis [23–25] and Sjögren’s syndrome [22], similar studies on radiographic or non-radiographic axSpA are scarce.

Given the cell specificity of miR expression profiles and considering the biological importance of CD4^+^ T cells and monocytes in the pathophysiology of the disease, we investigated and validated miR expression profiles in these two cell types from axSpA patients and controls in two independent cohorts, totaling 81 patients and 55 controls.

## Methods

### Patients

Two independent cohorts of 22 and 59 patients with axSpA were recruited from October 2014 to July 2017 in the Department of Rheumatology at Cochin Hospital in Paris, France. All patients fulfilled the 2009 ASAS classification criteria for axSpA [2]. Demographic and phenotypic data, HLA-B27 status, imaging (X-rays and/or MRI assessment of sacroiliac joints), and treatment history were collected. Disease activity was assessed using both the Bath Ankylosing Spondylitis Disease Index (BASDAI) and the Ankylosing Spondylitis Disease Activity Score (ASDAS) [26, 27]. All patients were TNF-blocker naïve or free of any biological treatment for more than 3 months.

**Fig. 1:**
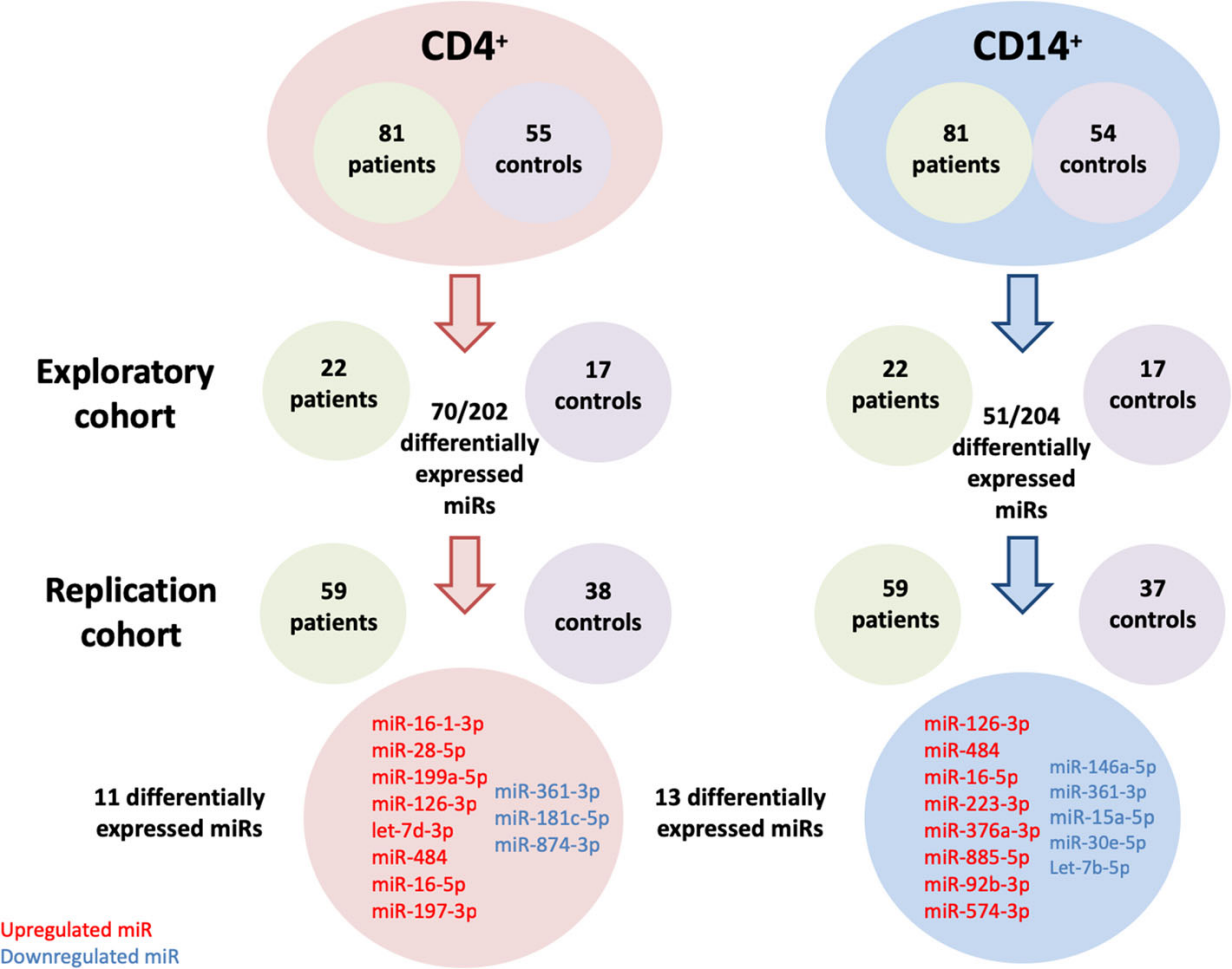
Description of the population studied and main results. The number of patients and controls with sufficient material used for the different analyses are shown in the graph. Seventeen parameters were investigated for association with the phenotype on the 81 patients (sex, age, disease duration, smoking status, HLA-B27, peripheral arthritis, enthesitis, uveitis, psoriasis, familial history of spondyloarthritis, radiographic sacro-iliitis, magnetic sacro-iliitis, CRP, BASDAI score, ASDAS, csDMARD intake, or NSAID intake). Differentially expressed miR expression were identified using a two-step process: Discovery in an exploratory cohort and confirmed in a replication cohort. MicroRNAs written in red are upregulated in the disease and those written in blue are downregulated in patients. A nominal *p* value < 0.05 was considered as significant

The exploratory cohort included 22 patients and 17 age- and sex-matched controls. The replication cohort included 59 patients and 38 age and sex-matched controls (Fig. 1). The main characteristics of patients and controls for both cohorts are shown in Table 1. Briefly, the mean age was 40 ± 13 years at enrolment, 73% were male, 77% were HLA-B27 positive, and 70% had radiographic sacroiliitis. The median disease duration from the onset of symptoms was 4 (0–46) years. Patients exhibited high disease activity with a mean BASDAI score of 49 ± 19 and a mean CRP of 12.5 ± 17 mg/l. Most patients were under NSAIDs at inclusion (80%), but few were treated with csDMARDs (15%).

**Table 1:** Demographic and disease-related characteristics of the population study

Variables	Overall population		Exploratory cohort		Replication cohort	
	Patients	Controls	Patients	Controls	Patients	Controls
No. of cases/controls	81	55	22	17	59	38
Male, *n* (%)	59 (73)	33 (62)	16 (72)	10 (59)	43 (73)	23 (64)
Age (years)^a^	40 ± 13	38 ± 8	41 ± 12	39 ± 9	39 ± 13	37 ± 8
Disease duration (years)^b^	4 (0–46)		6.5 (0.2–46)		4 (0–44)	
HLA-B27 positive, *n* (%)	58 (77)		16 (80)		42 (76)	
Current smoker, *n* (%)	38 (47.5)		11 (50)		27 (46)	
Clinical characteristics						
AxSpA, *n* (%)	78 (97.5)		22 (100%)		56 (96)	
AxSpa X-ray positive, *n* (%)	56 (70)		18 (82)		38 (65)	
AxSpA MRI positive, *n* (%)	63 (86)		18 (86)		45 (87)	
Peripheral arthritis	17 (21)		6 (27)		11 (19)	
Enthesitis	40 (50)		11 (50)		29 (50)	
IBD	4 (5)		0 (0)		4 (7)	
Uveitis	28 (35)		8 (36)		20 (34)	
Psoriasis	11 (14)		2 (9)		9 (16)	
Baseline CRP, mg/l^a^	12.5 ± 17		15 ± 24		12 ± 14	
BASDAI M0^a^	49 ± 19		42 ± 18		52 ± 19	
ASDAS M0^a^	3 ± 0.9		2.9 ± 0.8		3 ± 1	
Treatment						
NSAIDs, last 3 months, *n* (%)	64 (80)		19 (86)		45 (78)	
Steroids, last 3 months, *n* (%)	3 (3.75)		0 (0)		3 (5)	
csDMARDS, last 3 months, *n* (%)	12 (15)		7 (32)		5 (9)	
Methotrexate, *n* (%)	5 (42)		2 (29)		3 (60)	
Sulfasalazine, *n* (%)	7 (58)		5 (71)		2 (40)	

*IBD* inflammatory bowel disease, *CRP* C-reactive protein, *BASDAI* Bath Ankylosing Spondylitis Disease Activity Index, *ASDAS* Ankylosing Spondylitis Disease Activity Score

^a^Mean ± SD

^b^Median (max-min)

Controls were followed in the Department of Rheumatology for peripheral osteoarthritis without any history of inflammatory, infectious or tumoral disease; none had any immunosuppressive treatment. Written informed consent was obtained from all participants prior to enrolment, and the study was approved by the ethics committee Ile De France VII (number PP 14-039).

### Cell isolation and RNA isolation

PBMCs were isolated by density-gradient centrifugation using Unisep maxi+ tube (Eurobio, Courtaboeuf, France). Monocytes and CD4^+^ T lymphocytes were sorted from PBMCs by direct magnetic labeling with CD14^+^ and CD4^+^ microbeads according to the manufacturer’s instructions (Miltenyi Biotec, Paris, France). MicroRNAs were extracted using a 2-step protocol using both the RNeasy minElute Cleanup kit and the miRNeasy mini kit (Qiagen, Hilden, Germany) to obtain a smallRNA-enriched fraction according to the manufacturer’s protocol. Thirty nanograms of smallRNA were reverse transcribed into complementary DNA using the universal cDNA synthesis kit II following manufacturer’s protocol (Exiqon, Vedbaek, Denmark).

### Analysis of microRNA expression by real-time qPCR

The expression levels of 372 microRNAs were screened using the Human miRNome panel I (Version 4R, Exiqon, Vedbaek, Denmark) as previously described [22]. After quality controls and data processing steps (see Additional file 1), we retained only miRs that were expressed in at least 60% of the samples. Of the 372 miR assessed by real-time qPCR, 193 and 223 miR passed this threshold when analyzing monocytes and T lymphocytes together, 204 and 243 miRs passed this threshold in monocytes alone and 202 and 218 in CD4^+^ T lymphocytes in the exploratory and replication cohort, respectively. Only the differentially expressed microRNAs identified in the exploratory cohort were further analyzed in the 59 patients and 38 controls from the validation cohort (Fig. 1). MiR-155 and miR-146a were added to the analysis of the second cohort because of their relevant role in key inflammatory pathways and inflammatory diseases [20, 28–31].

### Comparison with results from previous work

We compared the identified panel of differentially expressed miR with data previously published in the literature. The Pubmed database was searched for articles in English analysing miR expression in spondyloarthritis with the following key words “microrna” or “miR” and “ankylosing spondylitis” or “spondyloarthritis” (until December 31, 2017). Only the miRs validated by qPCR in an independent cohort within the same publication and replicated by an independent group were included in the analysis.

### Statistical analysis

Analyses of differential expression and hierarchical clustering were performed using GenEx software (v.6, Exiqon). MiRs differentially expressed between axSpA patients and controls were identified using an unpaired *t* test with a nominal *p* value < 0.05 considered as significant. The study of miR expression in function of clinical parameters was performed on the 81 axSpA patients by combining the data from both cohorts using the R stat package (R Core Team (2017). R: A language and environment for statistical computing. R Foundation for Statistical Computing, Vienna, Austria). A Spearman’s test was used to study the correlation between miR expression levels and quantitative clinical parameters such as ASDAS, BASDAI, or CRP. The Mann-Whitney *U* test or Kruskall-Wallis test were used to test for differences in the distribution of miR expression between classes for categorical clinical parameters. Results were corrected for multiple testing using the Benjamini and Hochberg (BH) stratified false discovery approach. We considered results with a FDR-corrected *p* value < 0.05 as significant. The level of expression of miRs differentially expressed in CD14^+^ monocytes of SpA patients was compared between patients not treated with csDMARDs and patients treated with sulfasalazine or methotrexate patients using a three-way Kruskall-Wallis test.

KEGG pathway enrichment analysis was performed among target genes of each significantly differentially expressed miR in monocytes or CD4^+^ T lymphocytes separately using the DIANA tools [32]. Pathways were significantly enriched if the *p* value was lower or equal to 0.05 after correction for multiple testing, and the number of target genes belonging to this pathway was greater or equal to 5.

## Results

### MiR expression in patients and controls is cell type specific

One hundred sixty-three of the 193 miRs and 203 of the 223 miRs that could be reliably detected were significantly differentially expressed between CD4^+^ T lymphocytes and monocytes in the exploratory and replication cohort, respectively, after correction for multiple testing (data not shown). Principal component analysis of miR expression showed two main clusters corresponding to monocytes and T lymphocytes from both patients and controls. Hierarchical clustering revealed a lack of segregation between patients and controls within each cell type at this level of analysis (Additional file 1: Figure S1).

### MiR expression signatures in CD14^+^ monocytes and CD4^+^ T lymphocytes from axSpA patients

**Fig. 2:**
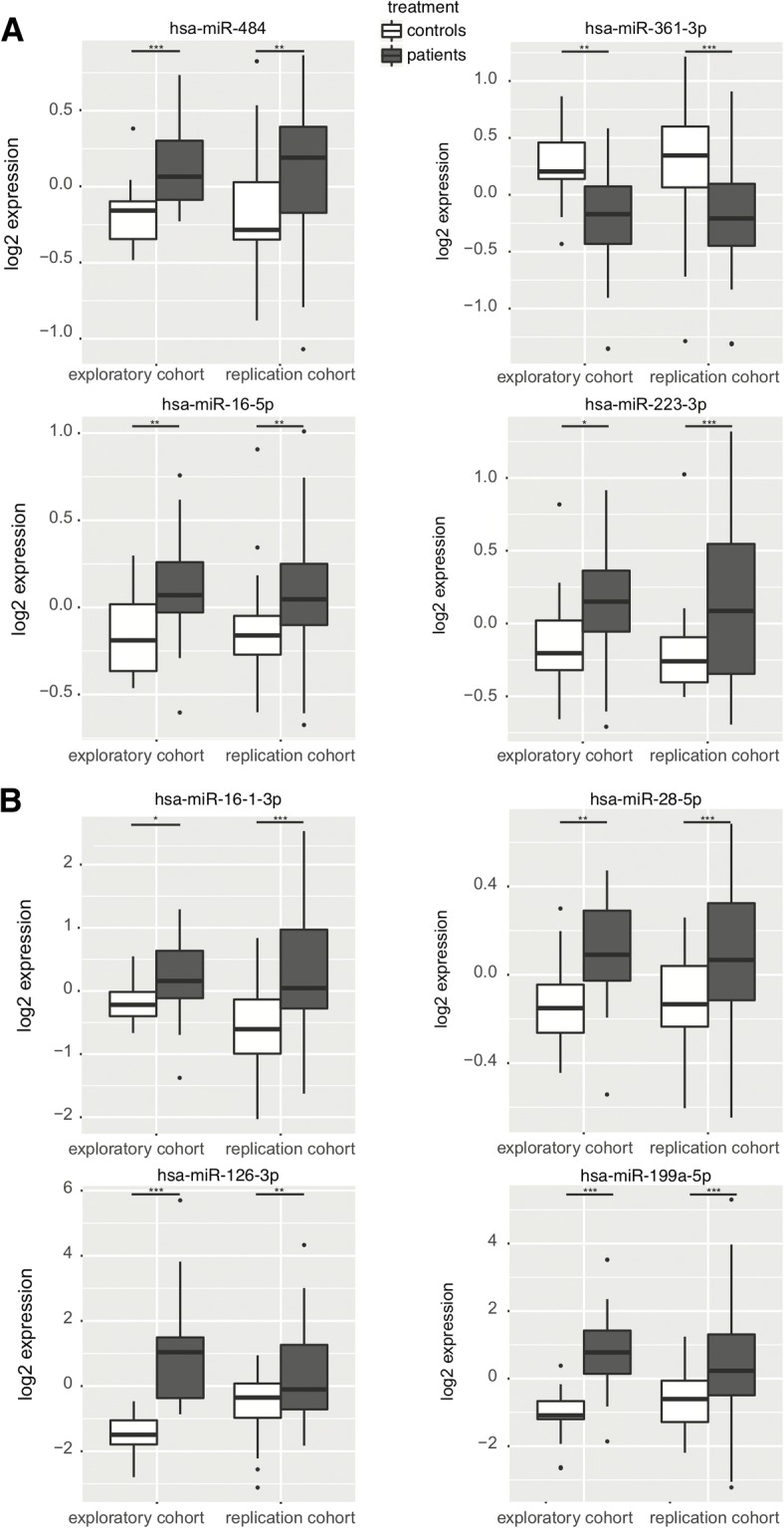
Distribution of the expression of the most differentially expressed microRNAs between axSpA patients and controls in CD14^+^ monocytes (**a**) and in CD4^+^ T lymphocytes (**b**) in the exploratory and replication cohorts. In monocytes, miR-484, miR-16-5p, and miR-223-3p were significantly upregulated and miR-361-3p was downregulated in patients (grey) compared to controls (white). In CD4^+^ T lymphocytes, the four most significantly deregulated miRs were upregulated in patients (grey) compared to controls (white). *p < 0.05, **p < 0.01, ***p < 0.001

In monocytes, 51 of the 204 detectable miRs were significantly differentially expressed in the exploratory cohort between patients and controls. In the replication cohort, we confirmed that 13 miRs were differentially expressed: 5 miRs were downregulated whereas 8 miRs were upregulated in monocytes from axSpA patients compared to controls (Table 2, Fig. 1). Among these miRs, miR-361-3p and miR-223-3p were the most significantly associated with the disease in the replication cohort (log2 fold change (log_2_FC): − 1.35, *p* = 5.80 × 10^− 5^ and log_2_FC: 1.27, *p* = 4.90 × 10^−4^, respectively) (Fig. 2a). The expression level of miR-146a-5p was significantly lower in monocytes from axSpA patients compared to controls (log_2_FC: − 1.31, *p* = 2.00 × 10^−3^).

**Table 2:** Differentially expressed microRNA in CD4^+^ or CD14^+^ cells between axSpA patients and controls

MicroRNA	Cell type	Log2 fold change^†^	exploratory cohort *P* value	replication cohort *P* value	Previously validated target genes*	Pathways**	Diseases
miR-16-1-3p	CD4^+^	1.85	0.04	1.30E−05	*IGF1R, CREBBP, EXT1/2*	Autophagy	
miR-28-5p	CD4^+^	1.15	3.06E−03	2.50E−04	*MAPK1, B7-H3*	Osteoblastic differentiation, JAK/STAT signaling pathway	
miR-199a-5p	CD4^+^	2.11	3.20E−06	8.90E−04	*CEBP/α, HIF-1α, IKKβ, NFkB1, EXTL3, SMAD4, WNT2*	TGF-β, monocyte to macrophage differentiation, osteoclast differentiation, WNT signaling	AS [43]
miR-126-3p	CD4^+^	1.74	6.57E−07	2.40E−03	*IκB, VCAM-1, LRP6, DNMT1, CXCL12*	NFκB, FcγR-mediated phagocytosis, HIF-1α signaling, PI3k/AkT signaling pathway	IBD [47], AS [40]
	CD14^+^	1.24	0.03	0.04			
let-7d-3p	CD4^+^	1.24	0.02	3.10E−03			
let-7b-5p	CD14^+^	− 1.21	0.01	0.02	*IFNβ1, TLR4, SOX9, IL17RC, ERAP2*	PI3k/AkT signaling pathway, WNT signaling, Apoptosis	CD [72], AS [41]
miR-361-3p	CD4^+^	− 1.26	1.11E−03	4.80E−03	*LRP5, FRIZZLED6, TRAF1, IL1RA*	WNT signaling	CD [72]
	CD14^+^	− 1.35	1.08E−03	5.80E−05			
miR-484	CD4^+^	1.2	5.24E−04	0.02	*IFNAR2, IL2, RUNX2*	Osteoblast differentiation	
	CD14^+^	1.22	9.89E−04	1.10E−03			
miR-16-5p	CD4^+^	1.11	3.91E−03	0.03	*WNT3A, LRP6, NFAT, SMAD3, BCL2, PDL-1, CASP1/5, IFNγ, EXT1/3, TNFAIP2, TGFB1, NFkB1, STAT3*	M1 macrophages polarization, T lymphocyte activation, TGF-β, PI3k/AkT and NOD pathways	AS [41, 73], RA [74]
	CD14^+^	1.16	4.98E−03	1.20E−03			
miR-197-3p	CD4^+^	1.52	3.77E−04	0.03	*SMAD7, IL1R1, PDCD4, IL18*	TGF-B, Osteoclast differentiation, Apoptosis	
miR-181c-5p	CD4^+^	− 1.23	7.41E−03	0.04	*IL2, CCL22, IL1α, BCL2*	Apoptosis, FcγR mediated phagocytosis, PI3k/AkT signaling pathway	
miR-874-3p	CD4^+^	− 1.18	5.27E−04	0.05	*STAT3, IKKβ*	Osteoblastic differentiation, FcγR mediated phagocytosis	
miR-223-3p	CD14^+^	1.27	0.05	4.90E−04	*NLRP3, CLDN8, FOXO3, IL6*	Monocyte survival, macrophage differentiation, NFκB pathway, Epithelial cell adhesion	IBD [62, 75], psoriasis [37], RA [74]
miR-146a-5p	CD14^+^	− 1.31	0.08	2.01E−03	*NFκB, TRAF6, IRAK1, TLR2/4, STAT1, RORα, MAP3K7*	NFκB and TNFα pathways, cytokines production, myeloid lineage differentiation	SpA [40, 76]
miR-15a-5p	CD14^+^	− 1.15	2.75E−04	4.60E−03	*BCL2, NFkB1, CARD8, TNFSF9, IKKα, SMAD7, IFNγ*	PI3k/AkT and NOD-like signaling pathways, BCR and TCR signaling, NFkB, TLR signaling, osteoclast differenciation, Apoptosis	
miR-376a-3p	CD14^+^	1.62	3.00E−06	6.05E−03	*CDK2, SMAD7, ATG4C, IL7*	Cell cycle, Bacterial invasion of epithelial cells, TGF-B pathway, Autophagy	
miR-885-5p	CD14^+^	1.42	5.57E−03	0.01	*CDK2, CASP16, EGF1R, CLDN1*	Cell cycle, Epithelial cell adhesion, Bacterial invasion of epithelial cells	
miR-574-3p	CD14^+^	1.35	1.78 E−03	1.89E−03	*RXRα, IRF4, EGFR*	Chondrogenic differentiation, Ubiquitin mediated proteolysis	FMF [77]
miR-92b-3p	CD14^+^	1.34	0.02	0.04	*SMAD2/5, BMP7, MAPK1*	Osteoblastic differentiation, TGF-B pathway	
miR-30e-5p	CD14^+^	− 1.18	9.06E−04	0.05	*BCL2, ATG12*	Apoptosis, autophagy	

*AS* ankylosing spondylitis, *CD* Crohn disease, *IBD* inflammatory bowel disease, *RA* rheumatoid arthritis, *FMF* familial Mediterranean fever

^†^In the replication cohort

*Experimentally validated target genes on miRWalk 2.0 website (http://zmf.umm.uni-heidelberg.de/apps/zmf/mirwalk2/) [78]

**Pathway analysis using DIANA-mirpath [32] or search on PubMed

In CD4^+^ T lymphocytes, 70 of the 202 detectable miRs were differentially expressed in the exploratory cohort. The analysis in the replication cohort confirmed 11 differentially expressed miRs, of which 3 were downregulated and 8 upregulated in CD4^+^ T lymphocytes from axSpA patients compared with controls (Table 2, Fig. 1). MiR-16-1-3p and miR-28-5p were the miRs most significantly associated with the disease (log_2_FC: 1.85, *p* = 1.30 × 10^− 5^ and log_2_FC: 1.15, *p* = 2.50 × 10^− 4^, respectively) (Fig. 2b). An upregulation of miR-484, miR-126-3p, and miR-16-5p and a downregulation of miR-361-3p were found in the two cell types in the same direction suggesting a broad impact on the immune response.

### Key pathways

The bioinformatic analysis using DIANA tools identified an enrichment of deregulated miRs in the extracellular membrane-receptor interaction pathway and fatty acid biosynthesis and metabolism (Additional file 1: Figure S2). Some miRs may be of particular interest because of their role in key pathways of the disease. Mir-199a-5p, mir-126-3p, mir-16-5p, mir-197-3p, mir-223-3p, mir-146a-5p, mir-15a-5p, mir-376a-3p, and miR-92b-3p are involved in the immune response through the NF-kB, TGFβ, and NOD pathways or by modulating monocyte activation or macrophage polarization (Table 2). Bone remodeling may be also altered by miRs regulating the osteoblastic or osteoclast differentiation or through the WNT signaling pathway. Finally, mir-223-3p, mir-885, and mir-376a-3p could act on the interplay between the microbiota and the immune response by regulating the permeability of the intestinal epithelium during bacterial invasion (Table 2).

### Analysis of relationships between miRNA expression and clinical characteristics of the patients

**Fig. 3:**
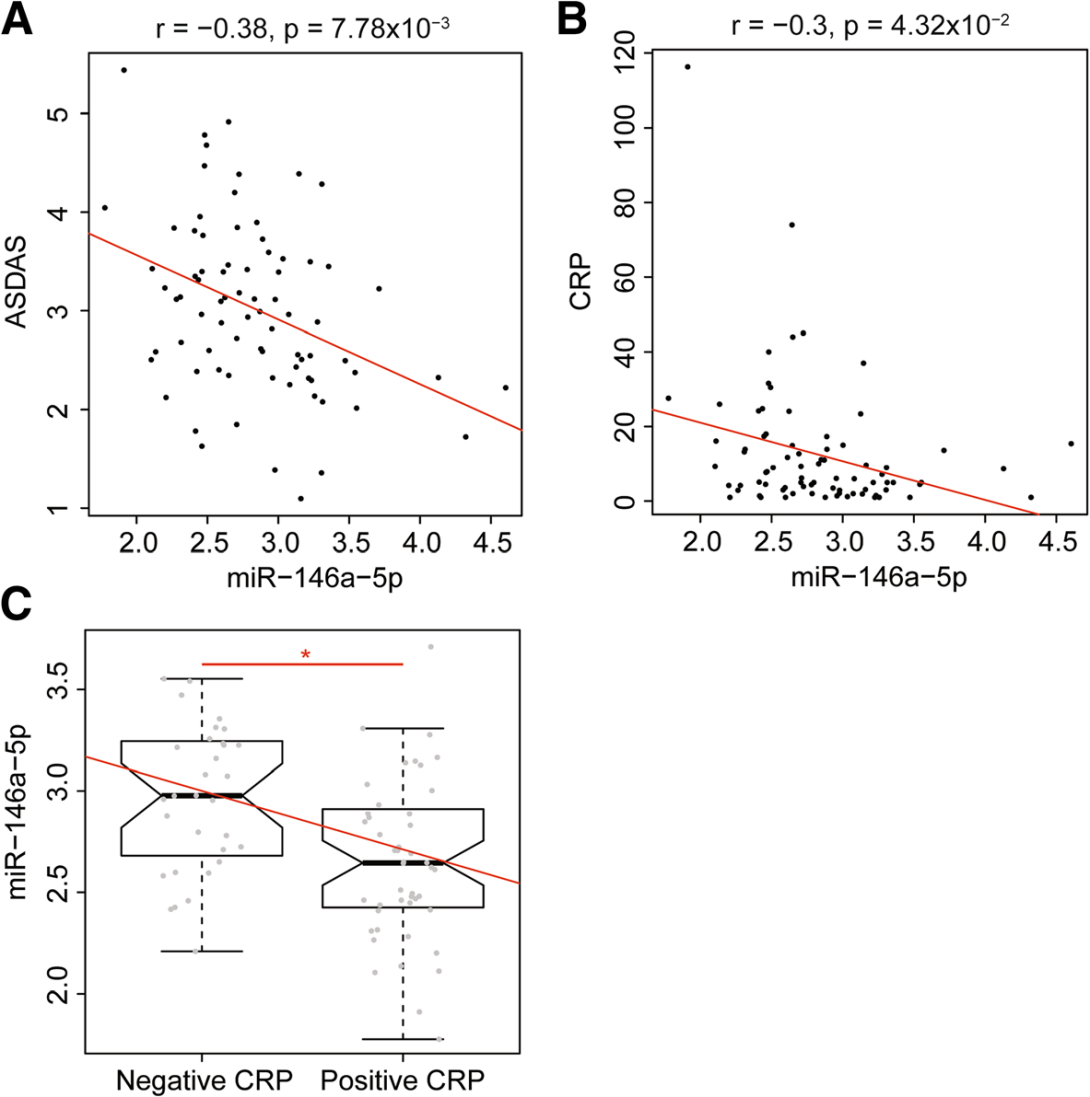
Association between the level of miR-146a-5p and the CRP level or ASDAS score in monocytes from axSpA patients. Negative correlation between the level of miR-146a-5p and **a.** the ASDAS score and **b.** the CRP levels, respectively. **c**. The miR-146a-5p level was significantly higher in patients who have never had a positive CRP value according to the laboratory threshold during the course of their disease. *p < 0.05

We further explored whether the differentially expressed validated miRs between patients and controls were associated with some specific patient’s characteristics. Twenty parameters were independently analysed covering clinical and biological parameters of the patients from both cohorts (Table 1). Inflammatory bowel disease (IBD) or corticosteroid intake was not included in the analyses as the effectives were not well-balanced for these two parameters. MiRs differentially expressed according to the disease’s phenotype with a nominal *p* < 0.05 are reported in Table 3. After Benjamini-Hochberg correction for multiple testing, only age, the ASDAS score, and the CRP value in CD14^+^ cells remained significantly associated with the expression of specific miRs. The level of miR-146a-5p was negatively associated with patients who had a positive CRP at least once in their disease history (log_2_FC: − 0.3; FDR = 0.02) and inversely correlated with the CRP levels (*r* = − 0.3; FDR = 0.04) and ASDAS score (*r* = − 0.40; FDR = 0.007) (Fig. 3). However, no association was found between any miR expression and the BASDAI score or the presence of a radiographic or magnetic sacro-iliitis. MiR-92b-3p was positively correlated with CRP value (*r* = 0.36, *p* = 0.01) and miR-126-3p and miR-574-3p were correlated with patients’ age (*r* = − 0.31, *p* = 0.05 and *r* = 0.29, p = 0.05 respectively). The use of NSAIDs did not impact miR expression while there was a trend toward a potential effect of csDMARD on three miRs (Table 3). When comparing patients not treated with csDMARDs to those treated with methotrexate (MTX) or sulfasalazine (SLZ), only miR-223-3p in CD14^+^ cells was found differentially expressed, with a decreased expression level in patients treated with sulfasalazine.

**Table 3:** Association between miRs expression and clinical or biological parameters in CD14^+^ and CD4^+^ cells from axSpA patients

Parameters	CD14^+^			CD4^+^		
	MicroRNA	*p* value	FDR corrected *p* value	MicroRNA	*p* value	FDR corrected *p* value
Age^2^	*miR-126-3p*	4.45E−03	*0.05*			
	*miR-574-3p*	8.45E−03	*0.05*			
	miR-146a-5p	0.04	0.18			
B27	miR-885-5p	0.03	0.46			
Male	miR-223-3p	0.03	0.37			
Disease duration^2^	miR-92b-3p	0.03	0.44			
Uveitis	miR-92-3p	0.02	0.42	miR-16-5p	0.05	0.49
Psoriasis	miR-15a-5p	0.01	0.10			
	miR-484	0.01	0.10			
Familial history of SpA	miR-126-3p	0.02	0.18	miR-16-1-3p	0.04	0.39
	miR-574-3p	0.03	0.18			
Axial involvement				miR-361-3p	0.04	0.28
Peripheral arthritis	miR-16-5p	0.03	0.43	miR-874-3p	0.04	0.45
Elevated CRP^1^	*miR-146a-5p*	1.82E−03	*0.02*	miR-361-3p	5.76E−03	0.06
	miR-361-3p	0.04	0.29			
CRP value^2^	*miR-92b-3p*	1.06E−03	*0.01*	miR-484	0.04	0.34
	*miR-146a-5p*	6.64E−03	*0.04*			
	miR-484	0.02	0.09			
	miR-574-3p	0.03	0.09			
BASDAI > 40	miR-219a-5p	0.03	0.21	miR-874-3p	0.04	0.39
	miR-361-3p	0.03	0.21			
BASDAI score^2^		0.01	0.14			
	miR-361-3p	0.05	0.30			
ASDAS score^2^	*miR-146a-5p*	5.98E−04	*7.78E−03*			
	miR-92b-3p	0.01	0.11			
Current smoker	let-7b-5p	0.05	0.30	miR-28-5p	0.01	0.11
	miR-223-3p	0.05	0.30			
csDMARDS	miR-376a-3p	0.02	0.09			
	miR-126-3p	0.02	0.09			
	miR-223-3p	0.01	0.09			

Only associations with a nominal *p* value < 0.05 are reported

MicroRNAs with a *p* value < 0.05 after Benjamin-Hochberg correction are written in italic

^1^At least one positive CRP value according to the laboratory reference during the disease course due to the disease’s activity

^2^Spearman test was used for quantitative values

### Comparison of the differentially expressed miRs between our results and previously published literature

To provide further evidence for a specific miR signature in SpA, we compared the validated miRs found deregulated in our study with previous reports on miRNA changes in axSpA. Of the initially identified 43 studies, 16 studies were finally retained (see Additional file 1: Figure S3 for details). When analysing results on monocytes and on T lymphocytes separately, 53 miRs were found deregulated in the disease in at least one study, but only 12 miRs were found in at least two studies. Three miRs were found consistently and significantly upregulated in patients compared to controls across different tissue samples: miR-126-3p, miR-484, and miR-92b-3p (Additional file 1: Figure S4). MiR-16-5p was found to be upregulated in two studies analysing T cells, including ours.

## Discussion

In this large-scale miR profiling study analysing two independent cohorts, we showed that patients with axSpA present solid evidence for a deregulation of specific miRs in monocytes (*n* = 13) and CD4^+^ T lymphocytes (*n* = 11). Only four miRs were consistently differentially expressed between patients and controls in both CD4^+^ and CD14^+^ cells, demonstrating two distinct specific signatures of miR expression profiles in monocytes and CD4^+^ T lymphocytes. The major strength of this study is the large number of miRs screened in sorted cells from a large number of clinically well-characterized subjects.

To date, miR expression profiles have been poorly studied in SpA. Two different approaches have been pursued: (i) the first strategy consisting in analysing miR expression in sorted cells and (ii) the second strategy consisting in analysing circulating miRs isolated from serum or plasma. The first approach is mainly performed to advance our understanding of cell type-specific mechanisms involved in disease pathophysiology; the second aims mainly at identifying biomarkers of the disease in easily accessible biological material for clinical routine. However, miR expression profiles obtained from one strategy cannot be extrapolated to the other, as multiple cell types and mechanisms contribute to the prevalence and abundance of miRs present as cell-free circulating miRs.

We decided to investigate the miR-expression profile of CD4^+^ T lymphocytes and monocytes because of their relevance for the pathophysiology of axSpA [3, 5, 9]. We found that the main factor influencing the miR expression profile is the cell type: almost 90% of the measured miRs were differentially expressed between CD4^+^ and CD14^+^ cells in our study, regardless of the disease status. Thus, the assessment of miR expression profiles in PBMCs or whole blood may be challenging as any observed changes could be related to variable cell subset composition, requiring thus the assessment of miR expression in sorted cells.

Some studies have addressed the impact of csDMARDs on miR expression in psoriasis or rheumatoid arthritis (RA). However, current data is conflicting. MiR-10a, miR-132-3p, miR-146a-5p, and miR-155-5p measured in whole blood or serum have been shown to be increased after MTX treatment in RA [33, 34] whereas miR-16 in plasma was not altered by the use of MTX in juvenile idiopathic arthritis [35]. In contrast, miR-146a measured in whole blood from patients with psoriasis was decreased after 3 months of methotrexate [36]. Also, in psoriasis, miR-223-3p was found downregulated in PBMC, but not altered in serum after the use of MTX and decreased in serum of patients treated by Etanercept [37, 38]. The authors suggested that the downregulation of miR-223 was not related to the treatment but rather to the immunosuppressive effect of the treatment on cells. We found only a single study reporting the impact of sulfasalazine on miR expression. Mir-21 was found to be negatively associated with *PDCD4* expression in whole blood from SpA patients not treated with csDMARDs, while positively associated with the expression of the same gene in patients treated with sulfasalazine [39]. In our study, we did not find miR-21 to be differentially expressed. Considering the presence of multiple cell types in most analyses, it is currently difficult to conclude if the use of csDMARDs has a direct influence on the miR expression profile or leads to a shift in cell populations. In our sorted cell populations, we found little evidence for the modification of the miRNA expression profiles by csDMARDs. However, our absolute number of patients treated with DMARDs were low, and larger studies will be required to fully address this question.

Several miRs found deregulated in CD4^+^ T cells or in monocytes in our study have been previously shown to be deregulated in other cell types in AS. Overexpression of mir-92b-3p, mir-16-5p, and mir-126-3p were also found overexpressed in T cells, fibroblasts, or PBMCs by others [40–42], reinforcing their likely involvement in the disease. No other study has focused on miR expression in monocytes from AS patients. Contrary to our result, Wang et al. found a lower expression of miR-199a-5p in T cells from 41 AS patients compared to healthy controls [43]. The main difference with our study is that miR expression was assessed on CD3^+^ T cells comprising both CD4^+^ and CD8^+^ T cells.

MiR-16-5p was found upregulated in both monocytes and T lymphocytes in our study and in CD3^+^ T lymphocytes from AS patients in a previous study [41]. It was assigned an anti-inflammatory function as it binds to the 3′-untranslated region (UTR) of *Tnfα* and *Il12b*, repressing their expression [44], or to the 3′UTR of *Pdcd4* (programmed cell death), resulting in a decreased level of Il-6 or Tnfα in mouse macrophages [45]. Nevertheless, miR-16 has also been shown to be able to create a pro-inflammatory environment [46].

The functional role of miR-126-3p has been well studied in IBD, where it was found upregulated in the colon tissue of patients with active ulcerative colitis [47, 48]. Furthermore, IκB, an inhibitor of NFκB, is a downstream target of miR-126-3p and the transfection of a miR-126-3p mimic decreases the expression of IκB in a dose-dependent manner [48]. MiR-126 also impairs intestinal mucosal barrier function under LPS stimulation by targeting *S1PR2* through the PI3k/AKT signaling pathway [49]. Functional experiments are required to further clarify its role in axSpA.

Macrophages play a major role in SpA as demonstrated by their abundant infiltration on biopsies from sacroiliac joints of AS patients, within the synovial tissue in chronic inflammatory arthritis, and in the gut mucosa [13, 50]. The macrophage infiltrate was shown to correlate with disease activity and to decrease after anti-TNF treatment [51, 52]. Macrophages represent a heterogeneous population of classically activated macrophages (M1), the main producers of inflammatory mediators such as TNFα, which are specialized in the clearance of pathogens and alternative M2 macrophages, which are involved in tissue repair and immunosuppression [53]. Whether M1 or M2 are causally implicated in SpA is still a matter of debate. CD163, a marker of M2 macrophages was found increased in the intimal lining layer of the synovial membrane and in gut mucosa from SpA patients [16, 50, 54]. In AS, the IFN-γ signature classically seen in M1 macrophages was impaired and the level of M1-derived cytokines was decreased in synovial fluid compared to patients with rheumatoid arthritis [17, 55]. However, these studies were focused on peripheral SpA and little is known regarding the role of M1 or M2 macrophages in axSpA. In PBMCs, a M2-like predominant polarization of monocytes has been reported, together with a M2/M1 ratio negatively correlated with CRP levels or BASDAI score [56]. A shift toward a M2 macrophage profile could facilitate bacterial survival and growth, especially for intracellular pathogens, such as Chlamydia or Yersinia, that have been involved in reactive arthritis and axial and peripheral arthritis in SKG mice [57, 58].

MiRs play a critical role in controlling macrophage differentiation [59]. MiR-16 was able to induce differentiation of mouse peritoneal macrophages into M1 macrophages from either the basal M0 or M2 polarized state and to activate purified CD4^+^ T lymphocytes, thus creating a pro-inflammatory environment [46]. In contrast, miR-124 and miR-223-3p were reported to contribute to M2 polarization or to limit M1 activation [60, 61]. The increased expression of miR-223-3p in our analysis suggests that circulating monocytes should be more prone to evolve into M2 macrophages in peripheral tissue. The function of miR-223-3p in SpA has never been addressed before, but it was found to be up-regulated in psoriasis and in inflammatory bowel disease [37, 62–64]. A dual role has been attributed to miR-223-3p in IBD: a protective role through the decreased in the release of inflammatory mediators and a facilitating role by increasing intestinal permeability. The overexpression of miR-223-3p in RAW 264.7 macrophages inhibited LPS-stimulated secretion of Il-6 or Il-1 by targeting *STAT3* [60]. In H. pylori-infected THP-1 monocytes, increased expression of miR-223-3p was correlated with decreased expression of NLRP3 [65]. MiR-223^−/−^ mice exhibit exacerbated DSS-induced colitis and an increased level of IL-1β through the loss of repressive function on *NLRP3* targeted by miR-223-3p [64]. MiR-223-3p may also increase the intestinal permeability by targeting *CLDN8* in response to IL-23 [62]. Therapeutic approaches in mice model have been elaborated targeting this dual role of miR-223. A systemic treatment with an antagomiR-223 was able to attenuate the phenotype in TNBS-induced colitis in mice [62]. By contrast, an intravenous nanoparticle delivery of a miR-223 mimic attenuated DSS-colitis in mice by targeting *Nlrp3* [64]. The upregulation of miR-223-3p among SpA patients in our study could both reflect the activated state of the monocyte/macrophages cells and an induced anti-inflammatory regulatory loop.

MiR-146a-5p is the most studied miR in autoimmune or inflammatory diseases. Also called anti-inflammoMir, miR-146a can bind to many targets involved in inflammatory signaling such as *IRAK1*, *TLR2/4* or *NFκB*. It acts as an effective regulator to prevent an overstimulated inflammatory state [31, 66]. IRAK1^−/−^ CD4^+^ T cells are less prone to produce IL17 under TGF-B and IL-6 stimuli and expressed a higher level of Foxp3 under TGF-B stimulation compared to WT CD4^+^ T cells [67]. Thus, IRAK1 can control the Th17/regulatory T-cells balance under specific stimuli, promoting Th17 differentiation. Keratinocytes from miR-146a^−/−^ mice were more sensitive to IL-17A stimulation that keratinocytes from WT mice, suggesting that mir-146a is a potent suppressor of IL17-driven disease [68]. MiR-146a-5p expression was found to be increased in skin biopsies and PBMCs from patients with psoriasis [69]. In SpA, discordant results have been published with an overexpression in fibroblasts and a decreased expression in PBMCs [70, 71]. We found a downregulation of miR-146a-5p in monocytes and its level was negatively correlated with ASDAS and CRP. Given its target genes, miR-146a-5p deregulation in monocytes might facilitate the polarization of Th17 cells from naïve CD4^+^ T cells through the release of pro-inflammatory cytokines.

## Conclusions

We have demonstrated a cell type-specific miRNA expression profile, whose deregulation could be related to the pathophysiology of the disease. More specifically, this study has highlighted some pathways that could be crucial in the onset or the maintenance of the disease such as the role of macrophage polarization, the impact of intestinal permeability or the modulation of the expression of pro-inflammatory cytokines.

## Additional files

Additional file 1: MicroRNAs expression by real-time qPCR. **Figure S1.** Principal component analysis of microRNAs expression and hierarchical clustering analysis in the exploratory cohort (A, C) and in the replication cohort (B, D). **Figure S2.** Pathways analysis of significant differentially expressed miRs in monocytes (A) and CD4^+^ T lymphocytes (B). **Figure S3.** Flow chart for the selection of published study for the comparison analysis. **Figure S4.** Cluster analysis of differentially expressed miRs in published studies from literature. (DOCX 2104 kb)

## Abbreviations

AS: Ankylosing spondylitis
ASDAS: Ankylosing Spondylitis Disease Activity Score
axSpA: Axial spondyloarthritis
BASDAI: Bath Ankylosing Spondylitis Disease Index
BH: Benjamini and Hochberg
CRP: C-reactive protein
csDMARD: Conventional synthetic disease modifying anti rheumatic drug
IBD: Inflammatory bowel disease
miR: MicroRNA
MTX: Methotrexate
NSAID: Non steroid anti-inflammatory drug
SLZ: Sulfasalazine
