# Supplementary material for: Deregulation of microRNA expression in monocytes and CD4+ T lymphocytes from patients with axial spondyloarthritis
Source: Arthritis Res Ther. 2019 Feb 12;21:51. doi: 10.1186/s13075-019-1829-7 (PMC6373047; doi:10.1186/s13075-019-1829-7)
Supplement: Supplementary file 1 — MicroRNAs expression by real-time qPCR. Figure S1. Principal component analysis of microRNAs expression and hierarchical clustering analysis in the exploratory cohort (A, C) and in the replication cohort (B, D). Figure S2. Pathways analysis of significant differentially expressed miRs in monocytes (A) and CD4+ T lymphocytes (B). Figure S3. Flow chart for the selection of published study for the comparison analysis. Figure S4. Cluster analysis of differentially expressed miRs in published studies from literature. (DOCX 2104 kb) [file 13075_2019_1829_MOESM1_ESM.docx]

**Supplementary methods**

**MicroRNAs expression by real time qPCR.**

cDNA was diluted one hundred times and mixed with the ExiLENT SYBR^®^ Green master mix (Exiqon) and distributed into 384-well Exiqon Human miRNome panel I (Version 4) plates using a TECAN Freedom EVO^®^ 150 robot. The design of the panels allowed the analysis of the expression of 372 miRNAs. UniSp6 RNA was spiked-in during reverse transcription as positive control. qPCR reactions were performed using the following conditions: 95°C for 10 minutes, 45 amplification cycles at 95°C, 10s, 60°C, 1 min, ramp-rate 1.6°C/s on a LightCycler480 V2 instrument (Roche Diagnostics, Meylan, France). Eighteen plates with less than 200 microRNAs with a Cq-value lower than 37 cycles were repeated. MiR data was analyzed using the GenEx software (version 6, Exiqon) following the data analysis guidelines. Briefly, data from each sample were combined and inter-plate calibration and quality control was performed. All miRNAs with Cq-values of more than 37 cycles or with less than 60% valid data were removed from the analysis. The Cq-values of all remaining miRs after the first steps of the process were normalized using the global mean expression of all miRs with a Cq-value less than 34 as validated previously [1]. Following normalization, the relative miR expression level was calculated using the 2^-∆∆Ct^ method [2]. DIANA-mirpath *(*[*http://diana.imis.athena-innovation.gr/DianaTools/index.php?r=mirpath*](http://diana.imis.athena-innovation.gr/DianaTools/index.php?r=mirpath)*)* was used for the pathway analysis of the differentially expressed miRs [3].

**Figure S1: Principal component analysis of microRNAs expression and hierarchical clustering analysis in the exploratory cohort (A, C) and in the replication cohort (B, D).** Each scatter-spot or each bar represents the average of normalized miRNA expression levels from CD4^+^ T lymphocytes of patients (blue) or controls (red) and CD14^+^ monocytes of patients (green) or controls (black).

**A** **B**


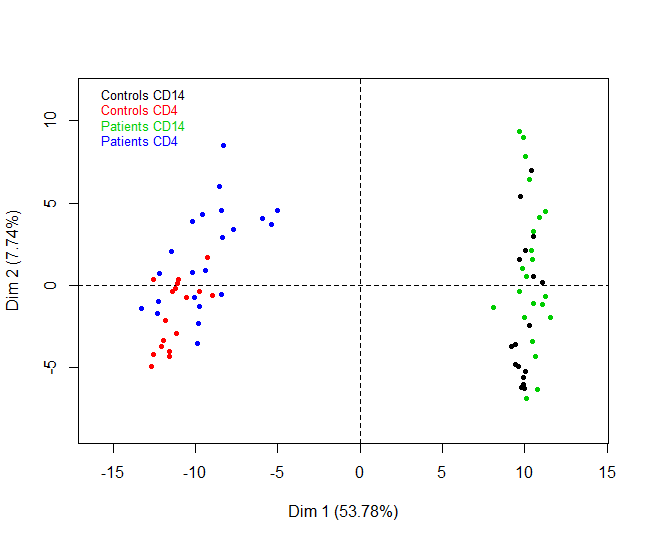
**
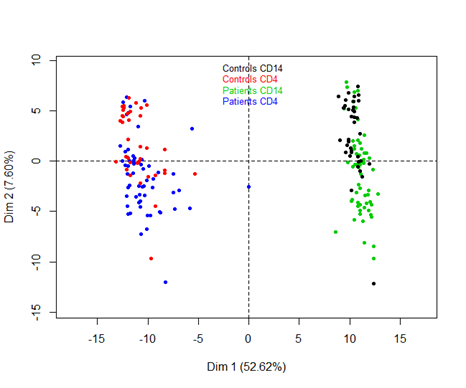
**

**C**


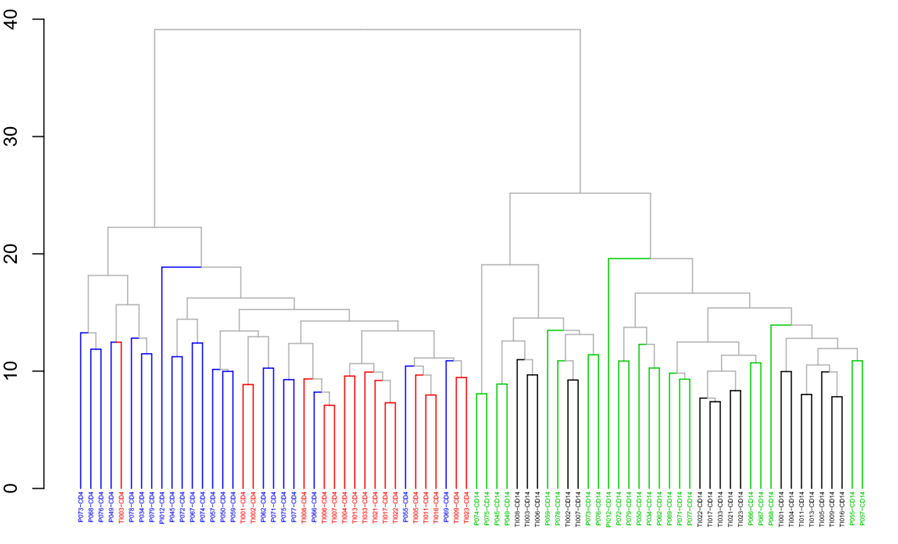


**D**

**
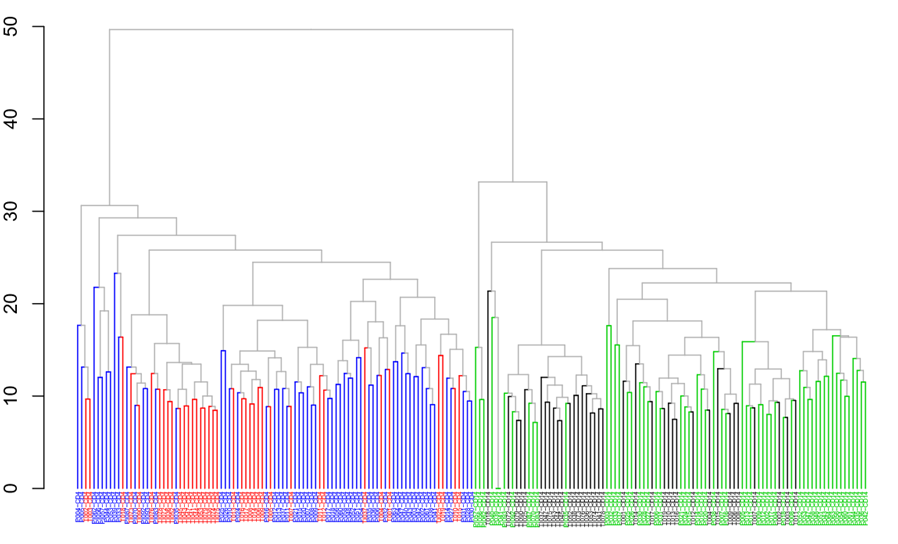
**

**Figure S2: Pathways analysis of significant differentially expressed miRs in monocytes (A) and CD4^+^ T lymphocytes (B).** An enrichment in fatty acid biosynthesis and metabolim and in ECM-receptor interactions were observed in both monocytes and T lymphocytes suggesting an involvment in the pathophysiology of the disease.

**A**

**
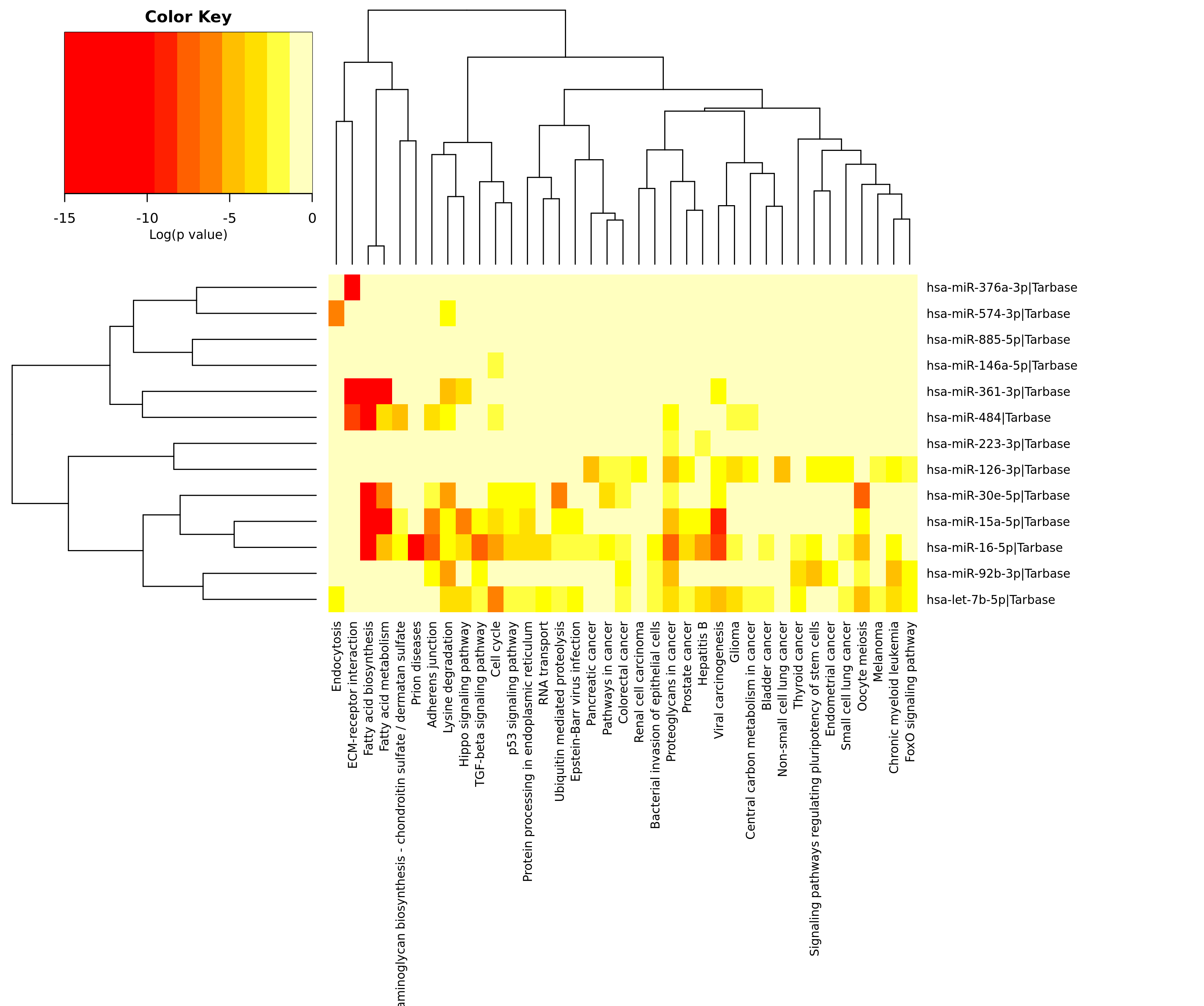
**

**B**

**
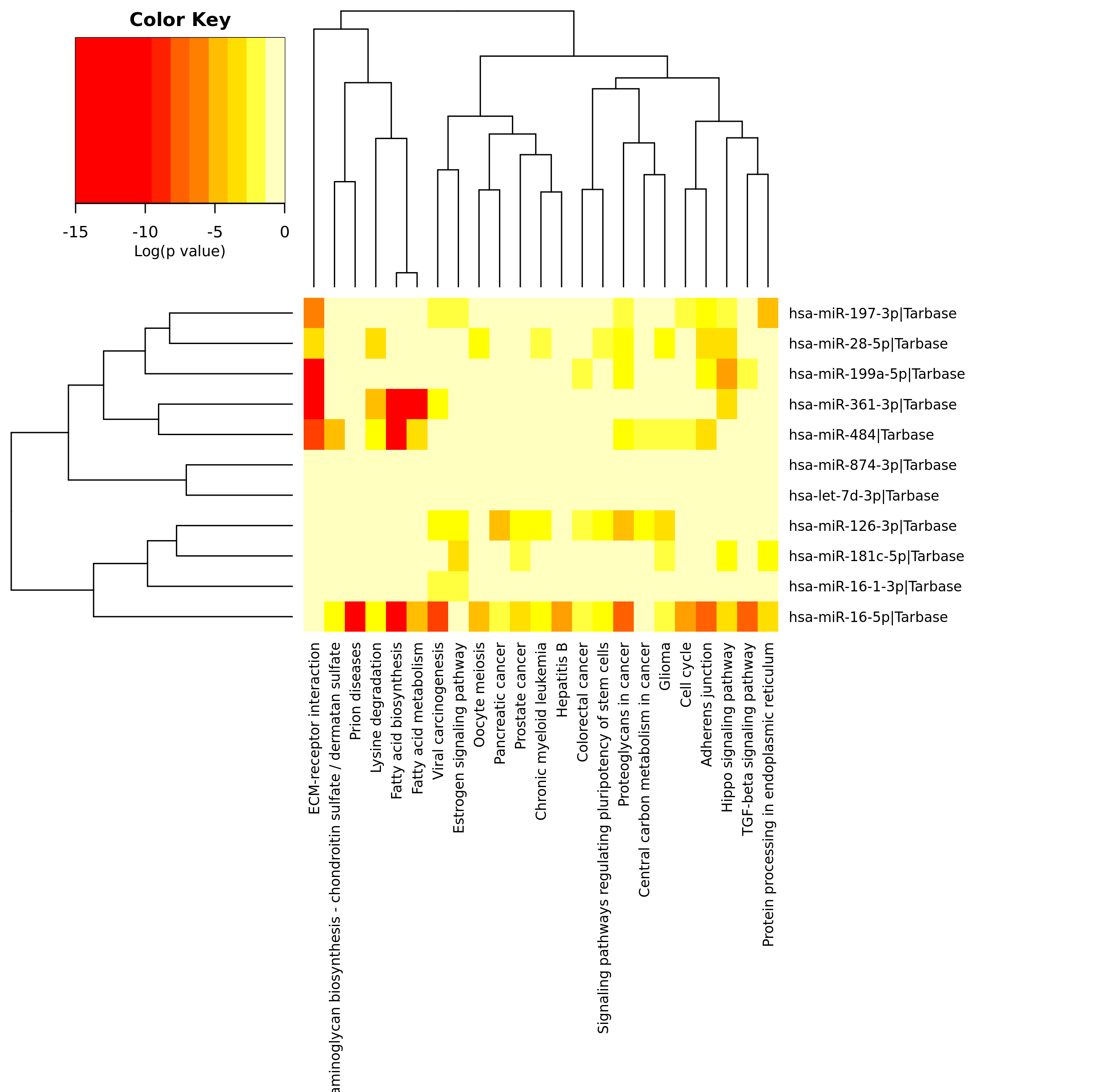
**

**Figure S3: Flow chart for the selection of published study for the comparison analysis.** Sixteen studies were retained and compared to the results of the presented study.

**
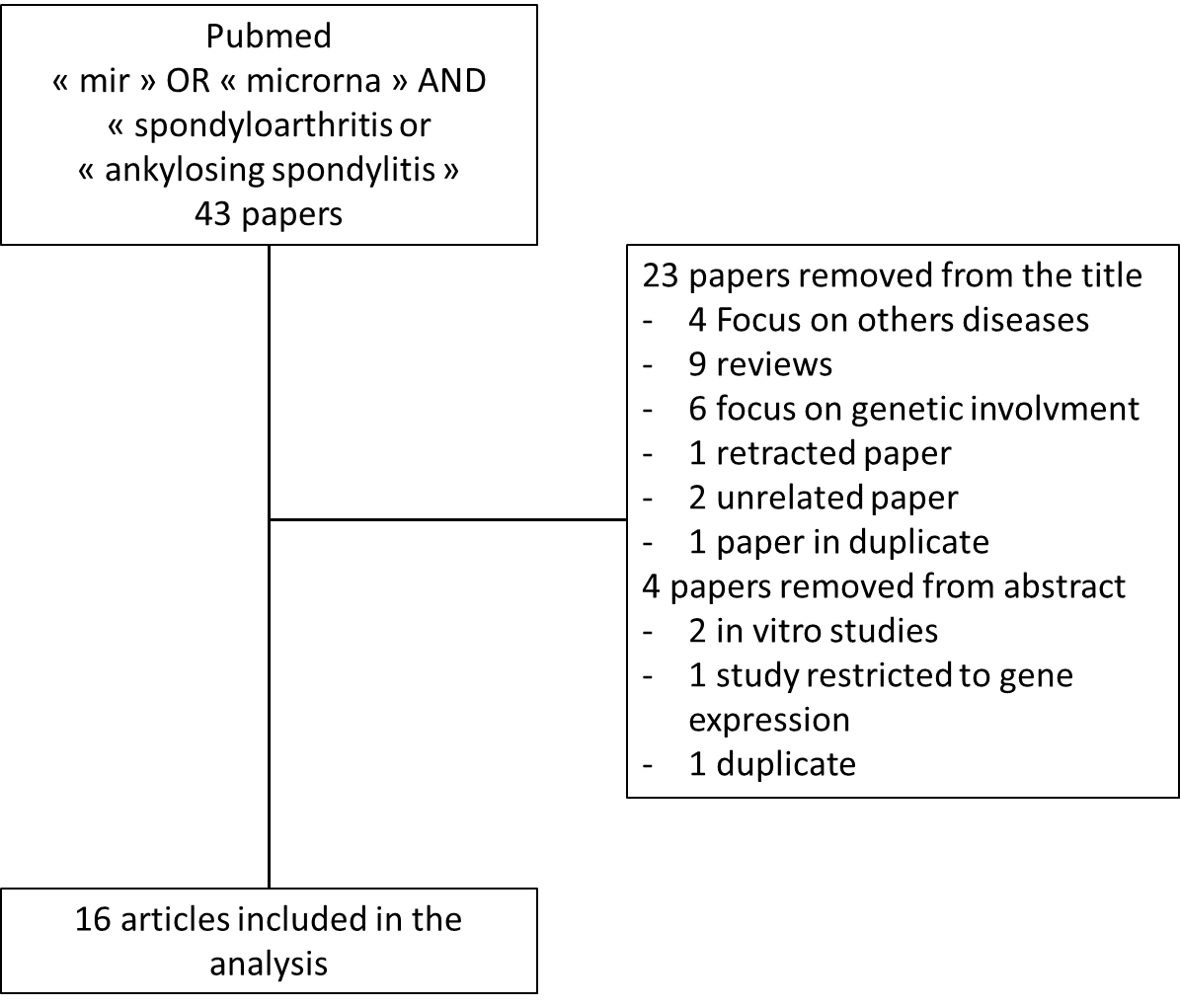
**

**figure S4: Cluster analysis of differentially expressed miRs in published studies from literature** [4–19]**.** Red colour represents miRs with lower expression levels in patients and blue colour represents overexpression of selected miRs in patients. MiRs differentially expressed in the same direction across studies are in bold. SFMC : synovial fluid mononuclear cell, PBMC: peripheral blood mononuclear cell.

**
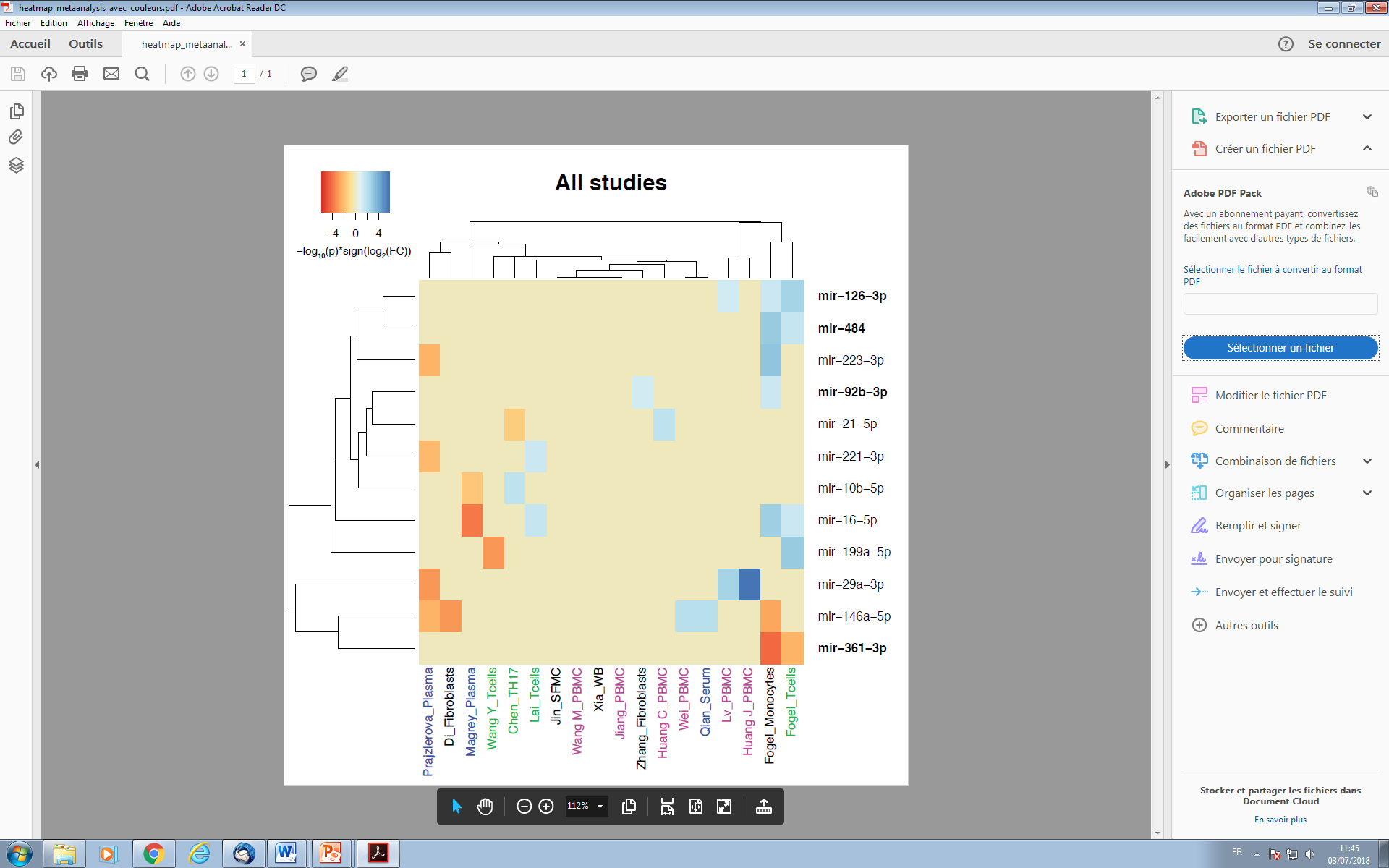
**

**References**

1. Mestdagh P, Van Vlierberghe P, De Weer A, Muth D, Westermann F, Speleman F, et al. A novel and universal method for microRNA RT-qPCR data normalization. Genome Biol. 2009;10:R64.

2. Livak KJ, Schmittgen TD. Analysis of relative gene expression data using real-time quantitative PCR and the 2(-Delta Delta C(T)) Method. Methods San Diego Calif. 2001;25:402–8.

3. Vlachos IS, Kostoulas N, Vergoulis T, Georgakilas G, Reczko M, Maragkakis M, et al. DIANA miRPath v.2.0: investigating the combinatorial effect of microRNAs in pathways. Nucleic Acids Res. 2012;40:W498-504.

4. Prajzlerová K, Grobelná K, Hušáková M, Forejtová Š, Jüngel A, Gay S, et al. Association between circulating miRNAs and spinal involvement in patients with axial spondyloarthritis. PloS One. 2017;12:e0185323.

5. Di G, Kong L, Zhao Q, Ding T. MicroRNA-146a knockdown suppresses the progression of ankylosing spondylitis by targeting dickkopf 1. Biomed Pharmacother Biomedecine Pharmacother. 2018;97:1243–9.

6. Magrey MN, Haqqi T, Haseeb A. Identification of plasma microRNA expression profile in radiographic axial spondyloarthritis-a pilot study. Clin Rheumatol. 2016;35:1323–7.

7. Wang Y, Luo J, Wang X, Yang B, Cui L. MicroRNA-199a-5p Induced Autophagy and Inhibits the Pathogenesis of Ankylosing Spondylitis by Modulating the mTOR Signaling via Directly Targeting Ras Homolog Enriched in Brain (Rheb). Cell Physiol Biochem Int J Exp Cell Physiol Biochem Pharmacol. 2017;42:2481–91.

8. Chen L, Al-Mossawi MH, Ridley A, Sekine T, Hammitzsch A, de Wit J, et al. miR-10b-5p is a novel Th17 regulator present in Th17 cells from ankylosing spondylitis. Ann Rheum Dis. 2017;76:620–5.

9. Lai N-S, Yu H-C, Chen H-C, Yu C-L, Huang H-B, Lu M-C. Aberrant expression of microRNAs in T cells from patients with ankylosing spondylitis contributes to the immunopathogenesis. Clin Exp Immunol. 2013;173:47–57.

10. Jin H-M, Cho Y-N, Kee S-J, Lee S-S, Park Y-W, Kim T-J. Micro-Ribonucleic Acid Profiles From Microarray in Ankylosing Spondylitis. Arch Rheumatol. 2016;31:121–6.

11. Wang M, Wang L, Zhang X, Yang X, Li X, Xia Q, et al. Overexpression of miR-31 in Peripheral Blood Mononuclear Cells (PBMC) from Patients with Ankylosing Spondylitis. Med Sci Monit Int Med J Exp Clin Res. 2017;23:5488–94.

12. Xia Y, Chen K, Zhang M-H, Wang L-C, Ma C-Y, Lin Y-L, et al. MicroRNA-124 involves in ankylosing spondylitis by targeting ANTXR2. Mod Rheumatol. 2015;25:784–9.

13. Jiang Y, Wang L. Role of histone deacetylase 3 in ankylosing spondylitis via negative feedback loop with microRNA-130a and enhancement of tumor necrosis factor-1α expression in peripheral blood mononuclear cells. Mol Med Rep. 2016;13:35–40.

14. Zhang C, Wang C, Jia Z, Tong W, Liu D, He C, et al. Differentially expressed mRNAs, lncRNAs, and miRNAs with associated co-expression and ceRNA networks in ankylosing spondylitis. Oncotarget. 2017;8:113543–57.

15. Huang C-H, Wei JC-C, Chang W-C, Chiou S-Y, Chou C-H, Lin Y-J, et al. Higher expression of whole blood microRNA-21 in patients with ankylosing spondylitis associated with programmed cell death 4 mRNA expression and collagen cross-linked C-telopeptide concentration. J Rheumatol. 2014;41:1104–11.

16. Wei C, Zhang H, Wei C, Mao Y. Correlation of the expression of miR-146a in peripheral blood mononuclear cells of patients with ankylosing spondylitis and inflammatory factors. Exp Ther Med. 2017;14:5027–31.

17. Qian B-P, Ji M-L, Qiu Y, Wang B, Yu Y, Shi W, et al. Identification of Serum miR-146a and miR-155 as Novel Noninvasive Complementary Biomarkers for Ankylosing Spondylitis. Spine. 2016;41:735–42.

18. Lv Q, Li Q, Zhang P, Jiang Y, Wang X, Wei Q, et al. Disorders of MicroRNAs in Peripheral Blood Mononuclear Cells: As Novel Biomarkers of Ankylosing Spondylitis and Provocative Therapeutic Targets. BioMed Res Int. 2015;2015:504208.

19. Huang J, Song G, Yin Z, Luo X, Ye Z. Elevated miR-29a expression is not correlated with disease activity index in PBMCs of patients with ankylosing spondylitis. Mod Rheumatol. 2014;24:331–4.
